# Prescribed opioids in primary care: cross-sectional and longitudinal analyses of influence of patient and practice characteristics

**DOI:** 10.1136/bmjopen-2015-010276

**Published:** 2016-05-13

**Authors:** Robbie Foy, Ben Leaman, Carolyn McCrorie, Duncan Petty, Allan House, Michael Bennett, Paul Carder, Simon Faulkner, Liz Glidewell, Robert West

**Affiliations:** 1 Leeds Institute of Health Sciences, University of Leeds, Leeds, UK; 2 Calderdale Metropolitan Borough Council, Halifax, UK; 3 Yorkshire & Humber Commissioning Support Unit, Bradford, UK; 4 Health and Social Care Information Centre, Leeds, UK

**Keywords:** Primary, Opioids, Prescribing, Variations

## Abstract

**Objectives:**

To examine trends in opioid prescribing in primary care, identify patient and general practice characteristics associated with long-term and stronger opioid prescribing, and identify associations with changes in opioid prescribing.

**Design:**

Trend, cross-sectional and longitudinal analyses of routinely recorded patient data.

**Setting:**

111 primary care practices in Leeds and Bradford, UK.

**Participants:**

We observed 471 828 patient-years in which all patients represented had at least 1 opioid prescription between April 2005 and March 2012. A cross-sectional analysis included 99 847 patients prescribed opioids between April 2011 and March 2012. A longitudinal analysis included 49 065 patient-years between April 2008 and March 2012. We excluded patients with cancer or treated for substance misuse.

**Main outcome measures:**

Long-term opioid prescribing (4 or more prescriptions within 12 months), stronger opioid prescribing and stepping up to or down from stronger opioids.

**Results:**

Opioid prescribing in the adult population almost doubled for weaker opioids over 2005–2012 and rose over sixfold for stronger opioids. There was marked variation among general practices in the odds of patients stepping up to stronger opioids compared with those not stepping up (range 0.31–3.36), unexplained by practice-level variables. Stepping up to stronger opioids was most strongly associated with being underweight (adjusted OR 3.26, 1.49 to 7.17), increasing polypharmacy (4.15, 3.26 to 5.29 for 10 or more repeat prescriptions), increasing numbers of primary care appointments (3.04, 2.48 to 3.73 for over 12 appointments in the year) and referrals to specialist pain services (5.17, 4.37 to 6.12). Compared with women under 50 years, men under 50 were less likely to step down once prescribed stronger opioids (0.53, 0.37 to 0.75).

**Conclusions:**

While clinicians should be alert to patients at risk of escalated opioid prescribing, much prescribing variation may be attributable to clinical behaviour. Effective strategies targeting clinicians and patients are needed to curb rising prescribing, especially of stronger opioids.

**Strengths and limitations of this study**

- Novel use of routine data to identify patient-level and practice-level characteristics associated with individual opioid prescribing trajectories.
- Findings based on a large sample of primary care practices and patients from diverse metropolitan populations.
- Identified associations cannot be assumed as casual.
- Exploratory work involving multiple statistical testing will have increased risk of type 1 errors and hence falsely positive associations.
- Detection and magnitude of associations will have been affected by imprecision and biases inherent in routinely recorded data.

## Background

There is international concern over rising trends in opioid prescribing,^1–9^ largely attributed to prescribing for chronic non-cancer pain.^10^ Despite known short-term efficacy of opioids in neuropathic and musculoskeletal pain, evidence is limited on longer term effects on pain, quality of life, functioning, tolerance and addiction.^11–14^ The degree of pain relief obtained for chronic pain is often of marginal clinical importance.^15^ Efficacy decreases with long-term opioid use, although dependence can make treatment withdrawal challenging.^16^ Prescribed opioids are associated with psychosocial problems, hospitalisation and, even for weaker opioids such as codeine, increased mortality.^17–20^ The ‘opioid epidemic’ in the USA has coincided with sharp increases in prescription opioid-related deaths and overdoses.^21^

There is concern that patients with chronic pain are inappropriately being moved up the WHO ‘analgesic ladder’, originally developed for cancer pain, without considering alternatives to medication.^22–24^ Over a third of one patient cohort eventually more than doubled their original dose of opioids while around 10% were prescribed potentially hazardous high doses.^23^

Previous research has identified patient characteristics associated with long-term opioid prescribing, which include female gender; increasing age; comorbidities; mental health disorders; and prescribed benzodiazepines, anticonvulsants or antidepressants.^2^ ^23^ ^25–29^ Strong or higher dose long-term opioid prescribing is associated with female gender, middle age, socioeconomic deprivation, care home residence, mood disorders, neuropathy, low back pain, polypharmacy, prior prescribing of weak opioids and nicotine dependence.^9^ ^23^ ^30^ ^31^ Initiation of opioid prescribing is associated with higher baseline levels of pain, psychological distress, substance misuse and unhealthy lifestyles.^2^ ^32–37^ However, most of these studies took place in selected patient groups, for example, trial participants or military veterans;^34^ ^37^ less is known about factors associated with such trajectories in typical primary care populations.

Long-term prescribing may follow hospitalisation but usually starts in primary care.^29^ There are well-documented variations in wider prescribing practice and safety in UK general practice.^38^ Structural factors such as practice list size and postgraduate general practitioner (GP) training status only marginally explain variations in high-risk prescribing (potentially inappropriate prescribing of drugs to patients vulnerable to adverse drug events)^39^ and none in strong opioid prescribing.^9^

We aimed to improve understanding of patient trajectories towards long-term and stronger opioid prescribing in a UK primary care population. We examined trends and analysed routinely recorded primary care population data to identify patient and general practice characteristics associated with long-term prescribing, stronger opioid prescribing, and stepping up or down between weaker and stronger opioids.

## Methods

### Study design and setting

First, we examined trends in opioid prescribing between 1 April 2005 and 31 March 2012 using routinely collected data from general practices in Leeds and Bradford, UK. Second, we conducted a cross-sectional analysis to identify patient and practice characteristics associated with long-term and stronger opioid prescribing (April 2011 to March 2012). Third, we undertook a longitudinal analysis to examine associations with stepping up to or down from stronger opioids (April 2008 to March 2012). Leeds and Bradford have a combined population of almost 1.3 million. Leeds is typical of UK cities in terms of social deprivation indices and demographics. Bradford is more socially deprived and ethnically diverse; one-fifth of the population is of Pakistani ethnic origin.^40^

### Study participants

There were 192 general practices in Leeds and Bradford in May 2012. We invited all 157 practices then using the SystmOne computerised record system (http://www.tpp-uk.com/products/systmone), which permitted centralised data collection, to participate. We used opt-out recruitment to reduce selection bias by facilitating general practices' agreement to share anonymised patient data. Practices not wishing to share data could opt out. Where practices had migrated from other record systems, we included only full year data from SystmOne to ensure data completeness for the periods covered.

We collected patient data in 12-month segments spanning April to March to ensure complete data for each Quality and Outcomes Framework (QOF) reporting period.^41^ The QOF is a performance management system whereby general practices are remunerated according to achievement of targets reflecting quality of care across four domains of clinical, organisational, patient experience and additional services. We extracted data on all patients registered with practices on 1st April who were prescribed an opioid at any point in that year. Within each 12-month segment, we excluded patients under age 18 at the start of each year, patients with a coded cancer diagnosis and patients with coded substance misuse or prescribed opioids normally used exclusively to treat drug addiction (methadone and sublingual buprenorphine).

### Variables

Our dependent variables comprised long-term prescribing of any opioid and at least one prescription of a strong opioid at any time point. We defined long-term use as a record of four or more opioid prescriptions within a given study year, assuming an average duration of 4 weeks supply for each prescription.^42–44^ We categorised opioid strength according to potency.^45^ The ‘weaker’ group included weak-to-moderate strength opioids and comprised codeine (with or without paracetamol or ibuprofen), dihydrocodeine (with or without paracetamol), tramadol, pethidine, meptazinol and tapentadol. The ‘stronger’ opioids comprised diamorphine, morphine, oxycodone, fentanyl, hydromorphone, buprenorphine (excluding preparations used for substance misuse), pentazocine, dipipanone and papaveretum. We defined stronger opioid use as at least one prescription of the latter group within the year.

Independent variables included patient-level and practice-level factors we hypothesised or recognised from earlier work to be associated with opioid prescribing.^2^ ^18^ ^25^ ^30^ ^32–37^ At the patient level, we examined demographic, lifestyle, illness, prescribing and health service utilisation variables. Demographic variables comprised age, gender and ethnicity. Lifestyle variables comprised alcohol use, smoking and body mass index (BMI). We assigned patients with clinical codes associated with alcohol use to one of two groups—problem drinker and not a problem drinker. The problem drinker group consisted of males recorded as drinking 50 units or more and females drinking 35 units or more per week. We also included those with clinical codes indicating heavy use or referral to alcohol services. We used the most recently recorded BMI in each year and assigned patients accordingly as underweight (BMI under 18.5), normal (18.5–25), overweight (25–30) and obese (over 30).

Illness variables included pain-associated conditions and clinical presentations, mental illness and comorbidity. We reviewed diagnostic codes and used an iterative process (involving BL, AH, RF and DP) to categorise codes into three groups which we hypothesised might affect GPs' prescribing behaviour: definitively diagnosed disease (eg, rheumatoid arthritis), clinical presentations without a definite diagnosis (eg, ‘knee pain’) and pain-associated syndromes with stronger psychosocial features (eg, fibromyalgia).

As a reflection of patient comorbidity and complexity, we assessed polypharmacy using the number of different repeat prescription medicines within a study year (1–4, 5–9 and 10 or more). We excluded prescriptions for dressings, stoma equipment and medically indicated food.

We assessed health service utilisation using general practice consultations, missed appointments and specialist referrals. We could not distinguish which type of healthcare provider patients consulted (eg, GP, practice nurse). We summed general practice consultations and missed appointments separately for each patient per annum and categorised them as low (<5), medium (between 5 and 10) and high (>10).

We categorised referrals by coded groups of destination specialty: diagnostic test (eg, X-ray), medicine, musculoskeletal, neurology, specialist pain management and surgery. We excluded codes used <100 times by all participating general practices over the study period.

General practice-level variables comprised registered patient list size, numbers of partners and salaried GPs, and training status—all recorded as of 1 April 2011. We used practice-level Index of Multiple Deprivation (IMD) scores. The IMD measures area deprivation and is determined for each patient on the list, where available, and then averaged over the practice. We used overall achievement in the QOF clinical domain as a proxy measure for overall quality of care.

One consequence of collecting data coded within each 12-month period is that we only captured clinical codes entered within that year. For example, we could capture whether a patient was coded as having an alcohol or mental health problem within the year but not if either was coded in earlier years. Our analysis was therefore restricted to incident or rerecorded diagnoses but did not include prevalent or previous diagnoses.

### Data sources

The West and South Yorkshire and Bassetlaw Commissioning Support Unit remotely extracted data from participating SystmOne practices. The Unit also supplied information on general practice characteristics.

### Analysis

For our cross-sectional study, we analysed only 2011–2012 data to examine short-term versus long-term and stronger versus weaker opioid prescribing. This was to avoid bias that would arise from studying later samples: when practices changed from one patient record system to another (SystmOne), they only migrated live patient records, so patients that were alive early in our study period but died before the practice changed records system would have been omitted from the study.

For our longitudinal analysis of stepping up or down between weaker and stronger opioids, we assembled a cohort of patients prescribed an opioid during each of 4 years (2008–2012) and prescribed at least one strong opioid during 1 or more but not all 4 years. We excluded patients prescribed stronger opioids throughout all 4 years from the step-up analysis because they had no opportunity to step up. We excluded patients prescribed only weaker opioids from the step-down analysis since they have no opportunity to step down. We did not examine stepping up from none to weaker opioids as we lacked complete records from prior years of patients not prescribed opioids. We used the final 4 years of data for the analysis on trajectories for pragmatic reasons as it delivered a sufficiently large data set of patients with complete data.

We initially calculated unadjusted ORs and then adjusted for confounders and clustering of patients within practices. We modelled associations with long-term and stronger opioid prescribing using multilevel logistic regression with a random effect for practice to account for clustering of patients within practices. We found that practices coded ethnicity into 79 different categories, making analysis potentially problematic. We therefore classified ethnicity into four crudely aggregated groups for cross-tabulation of ethnicity with opioid strength and duration: British, Pakistani, other and unknown. We included a random term for ethnicity for the logistic regression modelling, permitting use of all of the recorded ethnicity categories. We considered this to be an efficient and appropriate way to handle this variable. Thus, we considered patients clustered within practice and within ethnicity.

We sought a parsimonious multivariate model to ensure greater robustness of our findings. We fitted models to show effects adjusted for other covariates and excluded covariates if their effect was negligible and did not reach statistical significance at the 5% level. For adjusted ORs, if a covariate or factor was dropped to achieve a more parsimonious model, then no OR is reported.

Practice characteristics were strongly correlated with one other as expected, with the number of GPs increasing with list size. We also included practice IMD score in the model. Partly for simplicity of interpretation, and concerns over non-linearity, we treated all covariates as categorical variables (eg, practice IMD). We judged that any subsequent measurement error would be compensated for by greater ease of interpretation. We generally used an ‘unknown’ category for missing variables (eg, BMI). For variables with very few missing values (eg, age), we excluded patient records as increasing the number of parameters would not have been sufficiently compensated for by including larger numbers of patient records.

We anticipated an interaction between sex and age and accounted for this in the analysis. We handled age in three categories: 18–49, 50–64 and over 65 years.

All analyses conducted used R statistical software, specifically Revolution R Open V.3.2.0, with the lme4 library V.1.1.7 (R Core Team. R: A language and environment for statistical computing. Secondary R: A language and environment for statistical computing 2015. http://www.R-project.org/).^46^

### Study size

The sample size was originally based on the assumption that we would recruit at least 70% of the then estimated 185 practices using SystmOne (later found to be only 157 practices), that is, 148 practices and around 800 000 registered patients in total. We assumed the prevalence of repeat prescribing of (weaker and stronger) opioids was around 5%, and that a sample size of ∼600 000 patients over 18 years of age, even after allowing for clustering within practices, would provide an accuracy within 0.5%. This was based on an estimated design effect of 50 and calculating two SDs. We anticipated that an effective patient sample size of 12 000 would provide scope to robustly estimate patient-level parameters for covariates and factors. With 148 practices, we also anticipated good scope to examine practice-level covariates, so that our models could robustly estimate up to 14 practice-level covariates using the ratio of 10 observations per covariate.

## Results

### Participants

One hundred and eleven (70.7%) practices shared patient data. Participating practices had a higher median number of all registered patients compared with non-participants (7091 vs 5857; p=0.04), a higher mean number of GPs (5 vs 4; p=0.04) and similar median practice IMD (33.7 vs 33.1; p=0.77).

The number of practices with available data rose from 43 in 2005–2006 to 111 in 2011–2012 (table 1). The number of patients prescribed an opioid at least once rose from 26 249 (6.6% of total adult patients) to 99 847 (12.8%) over the same period, and covered a total of 471 828 patient-years. We observed 471 828 patient-years in which all patients included had received at least one opioid prescription over the 7-year period.

**Table 1:** Practices, patients and opioid prescribing over 2005–2012

Year	Number of practices	Number of patients	Number of patients prescribed any opioid at least once (%)	Number of patients prescribed a weaker opioid at least once (%)	Number of patients prescribed a stronger opioid at least once (%)	Opioid prescriptions per patient prescribed opioids	Weaker opioid prescriptions per patient prescribed opioids	Stronger opioid prescriptions per patient prescribed opioids	Percentage of stronger opioids prescribed to patients prescribed opioids
2005–2006	43	398 007	26 249 (6.60)	25 998 (6.53)	512 (0.13)	4.31	4.18	8.86	4.0
2006–2007	64	704 205	41 784 (5.93)	41 143 (5.84)	1284 (0.18)	4.56	4.36	8.88	6.0
2007–2008	76	723 810	52 826 (7.30)	51 753 (7.15)	2119 (0.29)	4.63	4.37	8.59	7.5
2008–2009	91	741 960	71 394 (9.62)	69 888 (9.42)	3288 (0.44)	4.69	4.36	9.22	9.1
2009–2010	100	757 796	85 022 (11.22)	83 108 (10.97)	4364 (0.58)	4.74	4.36	9.40	10.2
2010–2011	107	773 170	94 706 (12.25)	92 195 (11.92)	5668 (0.73)	4.91	4.46	9.90	12.1
2011–2012	111	781 528	99 847 (12.78)	96 959 (12.41)	6605 (0.85)	5.02	4.39	10.39	13.7

For the 2011–2012 cross-sectional analysis, data for almost all patients were complete. Data on gender were unrecorded and treated as missing for three patients. Some practice-level variables were unavailable for one practice with 469 patients registered.

Of the sample, 60 776 (60.9%) were female, 41 016 (41.1%) aged 18–49 years at the start of the year, 26 460 (26.5%) 50–64 years and 32 371 (32.4%) 65 years or over. Most were categorised as British (55 150; 55.2%), while 11 464 (11.5%) were Pakistani, 10 802 (10.8%) other and 22 431 (22.5%) of unknown ethnicity. Table 2 summarises other patient and practice characteristics. Having earlier excluded patients with a code for cancer or treatment for substance misuse, we categorised 37 508 (37.6%) as prescribed long-term opioids, equivalent to 4.8% of the adult practice population. A total of 6605 (6.6%) of the sample were prescribed stronger opioids, or 0.85% of adults.

**Table 2:** Patient and practice characteristics for cross-sectional analysis of associations with long-term and stronger opioid prescribing

	Four or more opioids prescriptions within year (long term)		Strength of opioid prescribed at any point within year	
	No (%)	Yes (%)	Weaker only (%)	Stronger (%)
Number of patients	62 339	37 508	93 242	6 605
*Patient demography*				
Female 18–49 yreas	19 455 (31.2)	5636 (15.0)	24 113 (25.9)	978 (14.8)
Female 50–64 years	8470 (13.6)	6802 (18.1)	14 239 (15.3)	1033 (15.6)
Female 65–100 years	9189 (14.7)	11 225 (29.9)	18 188 (19.5)	2226 (33.7)
Male 18–49 years	12 373 (19.8)	3549 (9.5)	15 104 (16.2)	818 (12.4)
Male 50–64 years	6561 (10.5)	4627 (12.3)	10 514 (11.3)	674 (10.2)
Male 65–100 years	6288 (10.1)	5669 (15.1)	11 081 (11.9)	876 (13.3)
Missing gender	3 (0)		3 (0)	
Ethnicity				
British	31 877 (51.1)	23 273 (62.0)	51 021 (54.7)	4129 (62.5)
Pakistani	8525 (13.7)	2939 (7.8)	11 226 (12.0)	238 (3.6)
Other	7718 (12.4)	3084 (8.2)	10 329 (11.1)	473 (7.2)
Unknown	14 219 (22.8)	8212 (21.9)	20 666 (22.2)	1765 (26.7)
*Patient morbidity, behaviour and healthcare use*				
Mental health problem	1626 (2.6)	1156 (3.1)	2546 (2.7)	236 (3.6)
Definitively diagnosed disease	7359 (11.8)	6170 (16.4)	12 346 (13.2)	1183 (17.9)
Clinical presentation without a definite diagnosis	23 112 (37.1)	12 714 (33.9)	33 471 (35.9)	2355 (35.6)
Pain-associated syndrome	310 (0.5)	205 (0.5)	466 (0.5)	49 (0.7)
Alcohol problem	672 (1.1)	475 (1.3)	1055 (1.1)	92 (1.4)
Smoking	13 474 (21.6)	7173 (19.1)	19 422 (20.8)	1225 (18.5)
Body mass index				
Overweight or obese	6573 (10.5)	3972 (10.6)	9972 (10.7)	573 (8.7)
Normal weight	3232 (5.2)	1510 (4.0)	4484 (4.8)	268 (4.0)
Underweight	251 (0.4)	149 (0.4)	357 (0.4)	43 (0.6)
Unknown	52 273 (83.8)	31 877 (85.0)	78 429 (84.1)	5721 (86.6)
Number of repeat prescriptions				
0	15 066 (30.2)	4375 (11.9)	18 999 (23.6)	442 (7.3)
1–4	22 567 (45.2)	14 450 (39.4)	35 023 (43.5)	1994 (32.8)
5–9	8152 (16.3)	9759 (26.6)	16 280 (20.2)	1631 (26.9)
10–56	4150 (8.3)	8054 (22.0)	10 201 (12.7)	2003 (33.0)
Unknown	12 404 (19.9)	870 (2.3)	12 739 (13.7)	535 (8.1)
Number of consultations				
0–6	21 530 (35.2)	8350 (22.8)	29 035 (31.7)	845 (14.0)
7–12	19 810 (32.4)	10 647 (29.0)	29 174 (31.8)	1283 (21.2)
13–366	19 689 (32.3)	17 677 (48.2)	33 451 (36.5)	3915 (64.8)
Unknown	1310 (2.1)	834 (2.2)	1582 (1.7)	562 (8.5)
Number of missed appointments				
0	39 295 (63.0)	22 556 (60.1)	58 532 (62.7)	3319 (50.2)
1–2	17 197 (27.6)	10 756 (28.7)	26 061 (28.0)	1892 (28.6)
3–29	4537 (7.3)	3362 (9.0)	7067 (7.6)	832 (12.6)
Unknown	1310 (2.1)	834 (2.2)	1582 (1.7)	562 (8.5)
Referrals				
Diagnostic testing	6207 (10.0)	4368 (11.6)	9794 (10.5)	781 (11.8)
Medical specialty	5446 (8.7)	4317 (11.5)	8856 (9.5)	907 (13.7)
Musculoskeletal specialty	9370 (15.0)	6926 (18.5)	15 009 (16.1)	1287 (19.5)
Neurology	1126 (1.8)	1046 (2.8)	1890 (2.0)	282 (4.3)
Specialist pain management	460 (0.7)	1135 (3.0)	1070 (1.1)	525 (7.9)
Surgical specialty	3824 (6.1)	2598 (6.9)	5877 (6.3)	545 (8.2)
Practice characteristics				
List size				
1500–6999	22 869 (36.7)	12 461 (33.2)	33 133 (35.5)	2197 (33.3)
7000–10 999	20 127 (32.3)	12 884 (34.4)	30 855 (33.1)	2156 (32.6)
11 000–25 112	19 343 (31.0)	12 163 (32.4)	29 254 (31.4)	2252 (34.1)
Practice Index of Multiple Deprivation				
7–26.7	21 518 (34.5)	13 248 (35.3)	32 251 (34.6)	2515 (38.1)
26.7–39.5	20 029 (32.1)	12 506 (33.3)	30 129 (32.3)	2406 (36.4)
39.5–57.5	20 792 (33.4)	11 754 (31.3)	30 862 (33.1)	1684 (25.5)
Number of GPs				
0–4	25 852 (41.5)	14 305 (38.1)	37 645 (40.4)	2512 (38.0)
5–9	18 129 (29.1)	11 607 (30.9)	27 757 (29.8)	1979 (30.0)
9–20	18 358 (29.4)	11 596 (30.9)	27 840 (29.8)	2114 (32.0)
Proportion of salaried GPs				
0–16.7%	20 779 (33.3)	12 422 (33.1)	30 807 (33.0)	2394 (36.2)
16.7–40%	23 030 (36.9)	14 097 (37.6)	34 759 (37.3)	2368 (35.8)
40% and over	18 172 (29.2)	10 878 (29.0)	27 222 (29.2)	1828 (27.7)
Unknown	358 (0.6)	111 (0.3)	454 (0.5)	15 (0.2)
Patients per GP				
707–1278	21 638 (34.7)	13 670 (36.4)	33 052 (35.4)	2256 (34.2)
1279–1558	19 744 (31.7)	12 603 (33.6)	29 847 (32.0)	2500 (37.8)
1559–4447	20 599 (33.0)	11 124 (29.6)	29 889 (32.0)	1834 (27.8)
Unknown	358 (0.6)	111 (0.3)	454 (0.5)	15 (0.2)
Proportion of ‘first five’ GPs				
0	34 016 (54.6)	20 291 (54.1)	50 462 (54.1)	3845 (58.2)
10–27.3%	10 762 (17.3)	6705 (17.9)	16 407 (17.6)	1060 (16.0)
27.3–66.7%	17 203 (27.6)	10 401 (27.7)	25 919 (27.8)	1685 (25.5)
Unknown	358 (0.6)	111 (0.3)	454 (0.5)	15 (0.2)
Teaching status	26 323 (42.2)	16 118 (43.0)	39 319 (42.2)	3122 (47.3)
Total QOF clinical domain points				
243–647	21 583 (34.6)	12 859 (34.3)	32 281 (34.6)	2161 (32.7)
648–657	21 630 (34.7)	13 052 (34.8)	32 330 (34.7)	2352 (35.6)
658–661	19 126 (30.7)	11 597 (30.9)	28 631 (30.7)	2092 (31.7)

GP, general practitioner; QOF, Quality and Outcomes Framework.

The longitudinal analysis included 17 165 patients prescribed any opioid in each of the 4 years considered. For the analysis of stepping up to stronger opioids, we excluded 810 patients prescribed stronger opioids for each of the first 3 years (2008–2011). Thus, there were 16 355 patients with three opportunities each to step up, or 49 065 patient-years. Of these, 32 223 (65.7%) were female, 12 354 (25.2%) aged 18–49 years, 16 343 (33.3%) 50–65 years and 20 866 (42.5%) 65 years or over. Most patient-years were British (25 821; 52.6%), while 8004 (16.3%) were Pakistani, 4844 (9.9%) other and 10 515 (21.4%) of unknown ethnicity.

For the analysis of stepping down to weaker opioids, we excluded those 14 918 patients prescribed weaker opioids in the first 3 years. After excluding 3 patients with incomplete data, there were 2244 patients, or 6732 patient-years, available for the step-down analysis. Of these, 4662 (69.3%) were female, 1583 (23.5%) were aged 18–49 years, 2142 (31.8%) were 50–64 years and 3007 (44.7%) were 65 years or over. Most patient-years were British (4160; 61.8%), while 480 (7.1%) were Pakistani, 4844 (9.9%) other and 1476 (21.9%) were of unknown ethnicity. Table 3 summarises sample characteristics for the longitudinal analyses.

**Table 3:** Patient and practice characteristics for longitudinal step-up and step-down analyses

	Stepping up to stronger opioids		Stepping down from stronger opioids	
	Yes (%)	No (%)	Yes (%)	No (%)
Number of patient years	1 662	47 403	937	5 795
*Patient demography*				
Female 18–49 years	255 (15.3)	8174 (17.2)	137 (14.6)	879 (15.2)
Female 50–64 years	317 (19.1)	9642 (20.3)	196 (20.9)	1181 (20.4)
Female 65–100 years	600 (36.1)	13 235 (27.9)	354 (37.8)	1915 (33.0)
Male 18–49 years	117 (7.0)	3808 (8.0)	45 (4.8)	552 (9.5)
Male 50–64 years	181 (10.9)	5705 (12.0)	92 (9.8)	673 (11.6)
Male 65–100 years	192 (11.6)	6839 (14.2)	113 (12.0)	625 (10.8)
British	1045 (62.9)	24 776 (52.3)	542 (57.8)	3618 (62.4)
Pakistani	119 (7.2)	7885 (16.6)	91 (9.7)	389 (6.7)
Other	157 (9.4)	4687 (9.9)	101 (10.8)	515 (8.9)
Unknown	341 (20.5)	10 174 (21.5)	203 (21.7)	1273 (22.0)
*Patient morbidity, behaviour and healthcare use*				
Mental health problem	72 (4.3)	1661 (3.5)	35 (3.7)	218 (3.8)
Definitively diagnosed disease	493 (29.7)	7493 (15.8)	186 (19.8)	1127 (19.4)
Clinical presentation without a definite diagnosis	900 (54.2)	18 366 (38.7)	384 (41.0)	2442 (42.1)
Pain-associated syndrome	21 (1.3)	341 (0.7)	8 (0.8)	55 (0.9)
Alcohol problem	17 (1.0)	1645 (3.5)	4 (0.4)	43 (0.7)
Smoking	194 (11.7)	5147 (10.8)	94 (10.0)	660 (11.4)
Body mass index				
Normal	43 (4.8)	1 192 (2.5)	25 (2.7)	166 (2.9)
Overweight or obese	118 (7.1)	3166 (6.7)	63 (6.7)	360 (6.2)
Underweight	9 (0.5)	85 (0.2)	0 (0)	18 (0.3)
Unknown	1 492 (90.0)	42 960 (90.6)	849 (90.6)	5251 (90.6)
Number of repeat prescriptions				
None	90 (5.4)	7836 (16.5)	98 (10.4)	431 (7.4)
1–4	495 (29.8)	19 609 (41.4)	318 (3.4)	1908 (33.0)
5–9	507 (30.5)	11 492 (24.2)	248 (2.6)	1594 (27.5)
10–56	570 (34.3)	8466 (17.8)	273 (29.1)	1862 (32.1)
Number of consultations				
0–6	117 (7.0)	11 122 (23.5)	132 (1.3)	800 (13.8)
7–12	298 (17.9)	14 415 (30.4)	224 (23.9)	1270 (21.9)
13–366	1247 (75.0)	21 866 (46.1)	581 (62.0)	3725 (64.3)
Number of missed appointments				
0	903 (54.3)	28 482 (60.0)	543 (58.0)	3302 (57.0)
1–2	564 (33.9)	14 267 (30.1)	297 (31.7)	1793 (30.9)
3–29	195 (11.7)	4654 (9.8)	97 (10.4)	700 (12.1)
Referrals				
Diagnostic testing	164 (9.9)	3384 (7.1)	80 (8.5)	491 (8.5)
Medical specialty	303 (18.2)	5200 (11.0)	141 (15.0)	863 (14.9)
Musculoskeletal specialty	470 (28.3)	6710 (14.2)	198 (21.1)	1100 (19.0)
Neurology	107 (6.4)	1073 (2.3)	22 (2.3)	249 (4.3)
Specialist pain management	220 (13.2)	919 (1.9)	47 (5.0)	494 (8.5)
Surgical specialty	186 (11.2)	3051 (6.4)	85 (9.1)	513 (8.8)
*Practice characteristics*				
List size				
1496–6999	21 563 (45.5)	679 (40.8)	2337 (40.3)	427 (45.6)
7000–11 000	14 959 (31.6)	563 (33.9)	2021 (34.9)	292 (31.2)
11 000–25 112	10 881 (23.0)	420 (25.3)	1437 (24.8)	218 (23.2)
Practice Index of Multiple Deprivation				
6.7–26.6	12 507 (26.4)	504 (30.3)	1711 (29.5)	267 (28.5)
26.7–39.4	16 270 (34.2)	669 (40.2)	2480 (42.8)	366 (39.1)
39.5–60	18 626 (39.3)	489 (29.4)	1604 (27.7)	304 (32.4)
Number of GPs				
0–4	18 496 (39.0)	566 (34.1)	1938 (33.4)	369 (39.4)
5–9	20 316 (42.9)	774 (46.6)	2638 (45.5)	416 (44.4)
10–20	8591 (18.1)	322 (19.4)	1219 (21.0)	152 (16.2)
Teaching status	18 803 (39.7)	763 (45.9)	2629 (45.4)	371 (39.6)
Total QOF clinical domain points within each year				
404–654	16 245 (34.3)	482 (29.0)	1550 (26.7)	275 (29.4)
655–677	15 569 (32.8)	544 (32.7)	1914 (33.0)	316 (33.7)
678–697	15 589 (32.9)	636 (38.3)	2331 (40.2)	346 (36.9)

GP, general practitioner; QOF, Quality and Outcomes Framework.

### Prescribing trends

The proportion of all patients prescribed a weaker opioid at least once increased over 7 years from 6.5% to 12.4%, while the proportion prescribed a stronger opioid at least once increased from 0.13% to 0.85% (table 1). For patients prescribed any opioids, the proportion prescribed stronger opioids increased from 4.0% to 13.7%.

### Cross-sectional analyses

The odds of long-term against shorter term opioid prescribing varied from 0.59 to 1.59 for practices, while those for stronger opioid prescribing varied from 0.32 to 2.32, suggesting major influences of practice on prescribing. None of the practice-level characteristics examined was associated with prescribing, except for practice IMD for long-term prescribing (1.21; 1.09–1.35; table 4).

**Table 4:** Cross-sectional analysis of associations with long-term and stronger opioid prescribing

	Four or more opioids prescriptions within year (long term)		Stronger opioid prescription at any point within year	
	Unadjusted OR (95% CI)	Adjusted OR (95% CI)	Unadjusted OR (95% CI)	Adjusted OR (95% CI)
*Patient demographics*				
Female 18–49 years	1	1	1	1
Female 50–64 years	2.77 (2.65 to 2.90)	**1.93 (1.83 to 2.02)**	1.79 (1.64 to 1.96)	**1.17 (1.06 to 1.29)**
Female 65–100 years	4.22 (4.05 to 4.39)	**2.39 (2.28 to 2.51)**	3.02 (2.79 to 3.26)	**1.46 (1.33 to 1.59)**
Male 18–49 years	0.99 (0.94 to 1.04)	**1.25 (1.18 to 1.32)**	1.34 (1.21 to 1.47)	**1.82 (1.64 to 2.02)**
Male 50–64 years	2.43 (2.32 to 2.55)	**1.75 (1.65 to 1.84)**	1.58 (1.43 to 1.75)	1.07 (0.96 to 1.19)
Male 65–100 years	3.11 (2.97 to 3.26)	**1.76 (1.67 to 1.86)**	1.95 (1.77 to 2.14)	**0.86 (0.78 to 0.96)**
British	1	Random effect	1	Random effect
Pakistani	0.47 (0.45 to 0.49)	Random effect	0.26 (0.23 to 0.30)	Random effect
Other	0.55 (0.52 to 0.57)	Random effect	0.57 (0.51 to 0.62)	Random effect
Unknown	0.79 (0.77 to 0.82)	Random effect	1.06 (1.00 to 1.19)	Random effect
*Patient morbidity, behaviour and healthcare use*				
Mental health problem	1.19 (1.10 to 1.28)	**1.12 (1.03 to 1.23)**	1.32 (1.15 to 1.51)	**1.23 (1.06 to 1.43)**
Definitively diagnosed disease	1.47 (1.42 to 1.53)	**1.09 (1.05 to 1.14**)	1.43 (1.34 to 1.54)	**1.12 (1.04 to 1.20)**
Clinical presentation without a definite diagnosis	0.87 (0.85 to 0.89)	**0.83 (0.81 to 0.86)**	0.99 (0.94 to 1.04)	–
Pain-associated syndrome	1.10 (0.92 to 1.31)	–	1.49 (1.11 to 2.00)	–
Body mass index				
Overweight or obese	1.01 (0.96 to 1.05)	–	0.79 (0.73 to 0.87)	**0.87 (0.79 to 0.95)**
Underweight	0.99 (0.81 to 1.21)	–	1.71 (1.24 to 2.34)	**1.89 (1.34 to 2.67)**
Number of repeat prescriptions				
1–4	2.21 (2.12 to 2.29)	**2.05 (1.96 to 2.14)**	2.45 (2.20 to 2.72)	**2.06 (1.85 to 2.30)**
5–9	4.12 (3.94 to 4.31)	**3.65 (3.47 to 3.84)**	4.31 (3.87 to 4.79)	**3.51 (3.13 to 3.95)**
10–56	6.68 (6.36 to 7.03)	**5.68 (5.35 to 6.03)**	8.44 (7.59 to 9.38)	**6.55 (5.81 to 7.38)**
Number of consultations				
0–6	1	1	1	1
7–12	1.39 (1.34 to 1.44)	**0.9 (0.86 to 0.93)**	1.51 (1.38 to 1.65)	1.1 (1.00 to 1.20)
13–366	1.37 (1.35 to 1.38)	**1.07 (1.03 to 1.12)**	4.02 (3.73 to 4.34)	**2.03 (1.86 to 2.21)**
Number of missed appointments				
0	1	1	1	1
1–2	1.19 (1.06 to 1.12)	**1.08 (1.04 to 1.12)**	1.28 (1.21 to 1.36)	**1.12 (1.06 to 1.20)**
3–29	1.29 (1.23 to 1.35)	**1.31 (1.24 to 1.39)**	2.08 (1.92 to 2.25)	**1.68 (1.53 to 1.84)**
Referrals				
Diagnostic testing	1.19 (1.14 to 1.24)	**0.95 (0.90 to 0.99)**	1.14 (1.06 to 1.24)	–
Medical specialty	1.36 (1.30 to 1.42)	**0.87 (0.83 to 0.92)**	1.52 (1.41 to 1.63)	–
Musculoskeletal specialty	1.28 (1.24 to 1.33)	**1.19 (1.14 to 1.24)**	1.26 (1.18 to 1.34)	–
Neurology	1.56 (1.43 to 1.70)	**1.27 (1.15 to 1.40)**	2.16 (1.90 to 2.45)	**1.52 (1.32 to 1.75)**
Specialist pain management	4.2 (3.76 to 4.68)	**3.7 (3.28 to 4.18)**	7.44 (6.68 to 8.28)	**5.74 (5.09 to 6.47)**
Surgical specialty	1.14 (1.08 to 1.20)	–	1.34 (1.22 to 1.47)	–
*Practice characteristics*				
List size				
1500–6999	1	–	1	–
7000–10 999	1.18 (1.13 to 1.21	–	1.05 (0.99 to 1.12)	–
11 000–25 112	1.15 (1.12 to 1.19)	–	1.16 (1.09 to 1.23)	–
Practice Index of Multiple Deprivation				
7–26.7	1	1	1	–
26.7–39.5	1.01 (0.98 to 1.05)	**1.17 (1.04 to 1.31)**	1.02 (0.97 to 1.09)	–
39.5–57.5	0.92 (0.89 to 0.95)	**1.21 (1.09 to 1.35)**	0.7 (0.66 to 0.75)	–
Number of GPs				
0–4	1	–	1	–
5–9	1.16 (1.12 to 1.19)	–	1.07 (1.01 to 1.14)	–
9–20	1.14 (1.11 to 1.18)	–	1.14 (1.07 to 1.21)	–
Proportion of salaried GPs				
Lower third	1	–	1	–
Middle third	1.02 (0.99 to 1.06)		0.88 (0.83 to 0.93)	–
Upper third	1 (0.97 to 1.03)		0.86 (0.81 to 0.92)	–
Patients per GP				
707–1278	1	–	1	–
1279–1558	1.01 (0.98 to 1.04)		1.23 (1.16 to 1.3)	–
1559–4447	0.86 (0.83 to 0.88)		0.9 (0.84 to 0.96)	–
Proportion of ‘first five’ GPs				
Lower third	1	–	1	–
Middle third	1.04 (1.01 to 1.08)	–	0.85 (0.79 to 0.91)	–
Upper third	1.01 (0.98 to 1.04)	–	0.83 (0.8 to 0.9)	–
Teaching status	1.03 (1.01 to 1.06)		1.23 (1.17 to 1.29)	–
Total QOF clinical domain points				
243–647	1	–	1	–
648–657	1.01 (0.99 to 1.04)		1.09 (1.02 to 1.15)	–
658–661	1.02 (0.99 to 1.02)		1.09 (1.03 to 1.16)	–

Bold typeface represent odds ratios for which the 95% confidence intervals did not cross 1.

GP, general practitioner; QOF, Quality and Outcomes Framework.

For ethnicity, the odds of long-term against shorter term prescribing varied from 0.65 to 1.64, based on 85 223 patients with complete data on covariates. Plots of practice residuals and ethnicity residuals were consistent with the assumption that the random effects followed Gaussian distributions for the long-term prescribing model. The ORs for stronger against weaker-only opioid prescribing ranged from 0.50 to 2.29, further indicating a strong association between ethnicity and prescribing.

Compared with women aged 18–49 years, long-term opioid prescribing was more likely in women aged 50–64 years (adjusted OR 1.93, 95% CI 1.83 to 2.02) as was stronger opioid prescribing (1.17, 1.06 to 1.29). These associations were respectively stronger in women 65 and over (2.39, 2.28 to 2.51; 1.46, 1.33 to 1.59).

Men below 50 years were more likely to be prescribed long-term opioids than their corresponding female age group (1.25, 1.18 to 1.32) with greater ORs for 50–64 years (1.75, 1.65 to 1.84) and 65 and over (1.76, 1.67 to 1.86). Males aged 18–49 were more likely (1.82, 1.64 to 2.02) and those 65 and over less likely to be prescribed stronger opioids (0.86, 0.78 to 0.96).

We found weak associations with coded diagnoses and problems. Long-term and stronger prescribing were associated with mental health problems (1.12, 1.03 to 1.23; 1.23, 1.06 to 1.23, respectively). Long-term and stronger prescribing were also associated with definitively diagnosed disease (1.09, 1.05 to 1.14; 1.04, 1.04 to 1.20, respectively), while long-term prescribing was inversely associated with clinical presentations lacking a definitive diagnosis (0.83, 0.81 to 0.86). Being overweight or obese was inversely associated (0.87, 0.79 to 0.95) whereas being underweight was associated with stronger opioid prescribing (1.89, 1.34 to 2.67).

Increasing polypharmacy was incrementally associated with long-term and stronger opioid prescribing, with adjusted ORs of 5.68 (5.35 to 6.03) and 6.55 (5.81 to 7.38), respectively, for those having 10 or more repeat prescriptions.

Relative to patients attending up to 6 primary care appointments over the year, those with 7–12 appointments were less likely to be prescribed long-term opioids (0.90, 0.86 to 0.93), while those attending 12 or more appointments were more likely to be prescribed long-term and stronger opioids (1.07, 1.03 to 1.12; 2.03, 1.86 to 2.21, respectively). Long-term and stronger prescribing were also both more likely if patients had missed booked primary care appointments, with the respective ORs rising from 1.08 (1.04 to 1.12) and 1.12 (1.06 to 1.20) for one to two missed appointments to 1.31 (1.24 to 1.39) and 1.68 (1.53 to 1.84) for three or more.

Long-term prescribing was less likely for patients referred for diagnostic tests (0.95, 0.90 to 0.99) and to general medicine (0.87, 0.83 to 0.92) and more likely for those referred to musculoskeletal services (1.19, 1.14 to 1.24). Long-term and stronger opioid prescribing were associated with referrals to neurology (1.27, 1.15 to 1.40; 1.52, 1.32 to 1.75, respectively) and specialist pain management services (3.70, 3.28 to 4.18; 5.74, 5.09 to 6.47, respectively).

### Longitudinal analyses

The odds of stepping up against not stepping up to a stronger opioid varied markedly from 0.31 to 3.36 by practice but less so, from 0.91 to 1.06, for stepping down. Practice-level variables explained none of the variation (table 5). There were variations by ethnic category, with ORs ranging from 0.55 to 1.40 for stepping up and 0.87 to 1.24 for stepping down. Compared with women aged 18–49 years, men aged 65 and over were less likely to step up to a stronger opioid (0.77, 0.63 to 0.94), while those aged 18–49 years were less likely to step down (0.53, 0.37 to 0.75).

**Table 5:** Longitudinal step-up and step-down analyses

	Stepping up to stronger opioids		Stepping down from stronger opioids	
	Unadjusted OR (95% CI)	Adjusted OR (95% CI)	Unadjusted OR (95% CI)	Adjusted OR (95% CI)
*Patient demography*				
Female 18–49 years	1	1	1	1
Female 50–64 years	1.05 (0.89 to 1.25)	0.94 (0.79 to 1.12)	1.07 (0.84 to 1.35)	1.07 (0.84 to 1.35)
Female 65–100 years	1.45 (1.25 to 1.69)	1.17 (1.00 to 1.38)	1.19 (0.96 to 1.47)	1.21 (0.97 to 1.50)
Male 18–49 years	0.99 (0.79 to 1.23)	1.21 (0.96 to 1.52)	0.55 (0.39 to 0.79)	**0.53** (**0.37 to 0.75)**
Male 50–64 years	1.02 (0.84 to 1.23)	0.97 (0.79 to 1.19)	0.88 (0.66 to 1.16)	0.90 (0.68 to 1.20)
Male 65–100 years	0.9 (0.74 to 1.09)	**0.77** (**0.63 to 0.94)**	1.16 (0.89 to 1.52)	1.20 (0.91 to 1.58)
British	1	Random effect		Random effect
Pakistani	0.36 (0.30 to 0.44)	Random effect	1.56 (1.22 to 2.00)	Random effect
Other	0.79 (0.67 to 0.94)	Random effect	1.31 (1.04 to 1.65)	Random effect
Unknown	0.8 (0.70 to 0.90)	Random effect	1.06 (0.90 to 1.27)	Random effect
*Patient morbidity, behaviour and healthcare use*				
Mental health problem	1.25 (0.98 to 1.59)	–	0.99 (0.69 to 1.43)	–
Definitively diagnosed disease	2.25 (2.02 to 2.50)	**1.64** (**1.46 to 1.85)**	1.03 (0.86 to 1.22)	–
Clinical presentation without a definite diagnosis	1.87 (1.69 to 2.06)	**1.60** (**1.43 to 1.79)**	0.95 (0.83 to 1.10)	–
Pain-associated syndrome	1.76 (1.13 to 2.74)	–	0.90 (0.43 to 1.89)	–
Alcohol problem	1.13 (0.69 to 1.84)	–	0.57 (0.21 to 1.60)	–
Smoking	1.09 (0.93 to 1.26)	–	0.87 (0.69 to 1.09)	–
Body mass index				
Normal	1	1	1	–
Overweight	0.93 (0.62 to 1.40)	0.95 (0.62 to 1.45)	1.05 (0.60 to 1.84)	–
Obese	1.13 (0.77 to 1.67)	1.08 (0.72 to 1.62)	1.31 (0.74 to 2.32)	–
Underweight	2.94 (1.39 to 6.22)	**3.26** (**1.49 to 7.17)**	–	–
Weight unrecorded	0.96 (0.71 to 1.31)	0.91 (0.66 to 1.25)	1.07 (0.70 to 1.65)	–
Number of repeat prescriptions				
None	1	1	1	1
1–4	2.2 (1.75 to 2.76)	**1.66** (**1.32 to 2.09)**	0.73 (0.57 to 0.94)	**0.71** (**0.55 to 0.91)**
5–9	3.84 (3.06 to 4.82)	**2.8** (**2.21 to 3.54)**	0.68 (0.53 to 0.89)	**0.66** (**0.51 to 0.86)**
10–56	5.86 (4.68 to 7.34)	**4.15** (**3.26 to 5.29)**	0.65 (0.50 to 0.83)	**0.60** (**0.46 to 0.77)**
Number of consultations				
0–6	1	1	1	–
7–12	1.97 (1.59 to 2.44)	**1.52** (**1.22 to 1.88)**	1.07 (0.85 to 1.35)	–
13–366	5.42 (4.48 to 6.56)	**3.04** (**2.48 to 3.73)**	0.95 (0.77 to 1.16)	–
Number of missed appointments				
0	1	1	1	1
1–2	1.25 (1.12 to 1.39)	–	1.01 (0.87 to 1.17)	–
3–29	1.32 (1.13 to 1.55)	–	0.84 (0.67 to 1.06)	–
Referrals				
Medical specialty	1.81 (1.59 to 2.06)	**1.17** (**1.02** **to** **1.34)**	1.01 (0.84 to 1.23)	–
Musculoskeletal specialty	2.39. 2.14 to 2.67)	**1.48** (**1.31 to 1.66)**	1.14 (0.97 to 1.36)	–
Neurology	2.97 (2.42 to 3.65)	**1.77** (**1.42 to 2.21)**	0.54 (0.34 to 0.83)	**0.59** (**0.38 to 0.91)**
Specialist pain management	7.72 (6.60 to 9.02)	**5.17** (**4.37 to 6.12)**	0.57 (0.42 to 0.77)	**0.61** (**0.45 to 0.83)**
Surgical specialty	1.83 (1.57 to 2.14)	**1.29** (**1.09 to 1.52)**	1.03 (0.81 to 1.31)	–
*Practice characteristics*				
List size				
1500–6999	1	–	1	–
7000–10 999	1.18 (1.13 to 1.21)	–	0.79 (0.67 to 0.93)	–
11 000–25 112	1.15 (1.12 to 1.19)		0.83 (0.7 to 0.99)	–
Practice Index of Multiple Deprivation				
7–26.7	1	–	1	–
26.7–39.5	1.01 (0.98 to 1.05)	–	0.95 (0.79 to 1.12)	–
39.5–57.5	0.92 (0.89 to 0.95)	–	1.22 (1.02 to 1.45)	–
Number of GPs				
0–4	1	–	1	–
5–9	1.16 (1.12 to 1.19)	–	0.83 (0.71 to 0.96)	–
9–20	1.14 (1.11 to 1.18)	–	0.66 (0.54 to 0.8)	–
Teaching status	1.03 (1.01 to 1.06)	–	1.03 (1.01 to 1.06)	–
Total QOF clinical domain points				
198–403	1	–	1	–
403–655	1.18 (1.04 to 1.33)	–	0.93 (0.78 to 1.11)	–
655–697	1.38 (1.22 to 1.55)	–	0.84 (0.70 to 0.99)	–

Bold typeface represent odds ratios for which the 95% confidence intervals did not cross 1.

GP, general practitioner; QOF, Quality and Outcomes Framework.

Stepping up was associated with coded definitive diagnoses (1.64, 1.46 to 1.85), clinical presentations lacking a definitive diagnosis (1.60, 1.43 to 1.79) and being underweight (3.26, 1.49 to 7.17). Increasing polypharmacy (4.15, 3.26 to 5.29 for 10 or more repeat prescriptions) was strongly associated with stepping up and inversely associated with stepping down (0.60, 0.46 to 0.77 for those with 10 or more prescriptions). Increasing numbers of primary care appointments were also associated with stepping up, with the OR reaching 3.04 (2.48 to 3.73) for over 12 appointments in the year.

Referrals to neurology and specialist pain management services were associated with stepping up (1.77, 1.42 to 2.21; 5.17, 4.37 to 6.12, respectively) and inversely associated with stepping down (0.59, 0.38 to 0.91; 0.61, 0.45 to 0.83, respectively). Referrals to general medicine (1.17, 1.02 to 1.34), musculoskeletal services (1.48, 1.31 to 1.66) and surgery (1.29, 1.09 to 1.52) were also associated with stepping up.

Table 6 provides an overall summary of our findings.

**Table 6:** Overall summary of associations with long-term and stronger opioid prescribing (+=adjusted OR of 1–2; ++=OR of 2–3; +++=OR over 3; italics=OR <1)

	Associations with long-term opioid prescribing	Associations with prescribing of stronger opioids	Associations with stepping up from weaker to stronger opioids	Associations with stepping down from stronger to weaker opioids
Age and gender	Female 50–64+ Female over 65++ Male (any age)+	Female 50–64+ Female over 65+ Male 18–49+ Male over 65−	Male over 65−	Male 18–49−
Ethnicity (fitted as random effect)	Pakistani, other and unknown ethnicity−	Pakistani and other ethnicity−	Pakistani, other and unknown ethnicity−	Pakistani and other ethnicity+
Morbidity and health behaviour	Mental health problem+	Mental health problem+		
	Definitive diagnosis+	Definitive diagnosis+	Definitive diagnosis+	
	Clinical presentation without definitive diagnosis−		Clinical presentation without definitive diagnosis+	
		Overweight or obese−	Underweight+++	
	Increasing polypharmacy+++	Increasing polypharmacy+++	Increasing polypharmacy+++	Polypharmacy−
Consulting behaviour	7–12 consultations per annum− 13 or over consultations per annum+	13 or over consultations per annum+	7–12 consultations per annum+ 13 or over consultations per annum+++	
	Increasing missed appointments+	Increasing missed appointments+		
Referrals	Diagnostic− Medicine− MSK+ Neurology+ Specialist pain management+++	Neurology+ Specialist pain management+++	Medicine+ MSK+ Neurology+ Specialist pain management+++ Surgery+	Neurology− Specialist pain management−
Practice IMD	Higher IMD+			

IMD, Index of Multiple Deprivation; MSK, musculoskeletal

## Discussion

Opioid prescribing in primary care has risen markedly, even after excluding patients with a code for cancer or drug dependence. The proportion of all patients prescribed a weaker opioid at least once almost doubled over 7 years, while the proportion prescribed a stronger opioid increased over sixfold. Much of this prescribing was to patients with unspecified non-malignant pain rather than to those with recorded diagnoses of a specific disease. We identified substantial, poorly explained variation among general practices in long-term and stronger opioid prescribing, particularly in the likelihood of stepping up to stronger opioids. Prescribing patterns varied by age, gender and ethnicity and suggested different risk groups; almost a third of all long-term opioid prescribing and of strong opioid prescribing occurred in women 65 and over. Younger men were particularly more likely to experience long-term and stronger opioid prescribing and less likely to step down once prescribed stronger opioids.

Our findings are based on a large sample of relatively unselected primary care patients from diverse UK metropolitan populations. While there is now a sizeable literature on risk factors for long-term or stronger opioid prescribing,^2^ ^9^ ^23^ ^25–27^ ^29^^–^31 less is known about patient trajectories. We found that stepping up from weaker to stronger opioids was most strongly associated with polypharmacy, more frequent primary care attendances, being underweight and pain service referrals. Patients were likely to step up regardless of whether or not painful conditions were definitively diagnosed, but those with no definitive physical diagnosis were less likely to be prescribed longer term opioids.

Our study has several limitations. First, we cannot imply causation from association. For example, patients with a coded mental health problem could be more likely to be prescribed an opioid or they could have developed a mental health problem as a result of chronic pain for which they were prescribed an opioid. Second, our exploratory work involved multiple statistical testing, increasing the risk of type 1 errors and hence falsely positive associations. Third, we recognised the potential for misclassification bias from our use of routinely recorded and coded data. For example, although musculoskeletal presentations to GPs are recorded in patient notes in 85% of such encounters, they are only clinically coded 32% of the time.^47^ Under-recording may have been more likely for diagnoses and clinical findings associated with stigmatisation (eg, mental illness, alcohol misuse). We anticipated that such imperfectly and incompletely coded data would reduce the identification and strength of any true associations. However, we sought typically recorded data in a typical sample of practices to enhance generalisability to other UK general practices and the utility of our findings. Any subsequent intervention to change prescribing practice based on searches of patient records would need to work in ‘real-world’ practices rather than those selected for better quality coding. Further imprecision was caused by our use of practice-level rather than patient-level deprivation. Fourth, we used data from only one practice computerised record system. Practice use of SystmOne is associated with higher QOF achievement; it is not known whether particular system characteristics facilitate higher quality of care, better data recording or both.^48^ This may be of limited relevance to our study as our dependent variables were not directly related to QOF targets. Fifth, the detection and magnitude of associations concerning diagnostic codes was reduced by ascertainment bias because we could only capture diagnoses and events recorded within each 12-month period. Subsequently, the strengths of associations related to mental health problems, alcohol problems and pain-related diagnoses were less than those found in other studies.^2^ ^27^ ^30^ ^33^ ^35^ ^37^ Conversely, including ‘ever-coded’ problems would have overestimated current diagnoses. Sixth, our models excluded patients with missing covariates; patients with missing data may systematically differ from those with complete data. Seventh, model selection was achieved through backwards stepwise selection, with careful attention paid to collinearity. This may have led to a model with optimistic goodness-of-fit characteristics (the R^2^ value will be too high) and for exaggerated coefficients that would be improved by shrinkage. These issues are unlikely to substantially alter the conclusions of our exploratory work. Eighth, participating practices were likely to be larger and have more primary care physicians than non-participating practices. Although smaller practice list size is associated with higher levels of high-risk prescribing,^39^ our adjusted analyses did not find a relationship between practice size and opioid prescribing. Ninth, the smaller sample size limited the precision of the step-down analysis and ability to detect associations of modest magnitude.

The prescribing patterns we found reflect varying interactions between population need and one or more of patient, clinician and health system behaviour. In women, long-term and stronger opioid prescribing increased with age, probably reflecting increasing population burden and impact of musculoskeletal disorders. The relative risk of stronger opioid prescribing was highest in younger men whereas older men were less likely to step up to and be prescribed stronger opioids. Younger men are prone to riskier and more severe accidental injuries.^49^ However, our finding that they were less likely to step down from stronger to weaker opioids is also compatible with an explanation of under-recognised or under-recorded opioid dependence.

There were consistent and large variations in prescribing by ethnicity. Patients coded as Pakistani or other ethnicities were particularly less likely than those coded as British to be prescribed long-term and stronger opioids, less likely to step up and more likely to step down. This is harder to explain according to population need and suggests a range of patient and clinician influences, including patient–clinician interactions.

We examined different ways of coding painful conditions to explore the influence of clinician certainty about diagnosis. Many musculoskeletal presentations are self-limiting but those of longer durations eventually acquire a diagnostic label. This is reflected in our finding that long-term and stronger opioid prescribing and stepping up were all associated with definitively diagnosed conditions (eg, osteoarthritis). Long-term prescribing was less likely for clinical presentations without a definitive diagnosis (eg, knee pain); the association with stepping up in this group may partly reflect additional symptomatic coding for exacerbations of definitively diagnosed conditions. However, we found no such associations for the smaller group of patients categorised as having pain associated syndromes with stronger psychosocial features (eg, fibromyalgia). The wider CIs suggest type 2 error but this finding may also reflect clinician reluctance to prescribe opioids without an obvious physical explanation. We could not determine how much opioid prescribing occurred before or as a consequence of specialist referrals; however, the association with referrals to neurology suggest that patients and GPs may struggle to find explanations for pain symptoms.

The association with coded mental health problems is consistent with an explanation that opioid prescribing occurs in response to psychological distress while also causing further dysphoria.

Some findings can only invite more speculative explanations. Being underweight was associated with stepping up to stronger opioids. This may reflect deterioration of long-term conditions, anorexia associated with taking stronger opioids or omitted codes for end-of-life care or drug dependence.

We found strong associations between increasing polypharmacy, which reflects multimorbidity, and all of long-term, stronger and stepping up opioid prescribing. Multimorbidity affects over a fifth of patients in primary care;^50^ osteoarthritis and cardiometabolic conditions (eg, hypertension, diabetes) are the mostly commonly paired.^51^ This complicates clinical management, such as prescribing decisions,^52^ and presents challenges for planning care, especially given that an average consultation covers 2.5 problems in 11.9 min.^47^ While patterns of opioid prescribing may partly reflect perceived clinical need, decisions to prescribe stronger opioids are often made reactively without explicitly assessing addiction risk or clear action planning.^53^

Only practice-level deprivation partly accounted for marked variation in prescribing patterns between general practices. Differences among practices in approaches to prescribing cannot be attributed to routinely recorded organisational characteristics such as the number of GPs in each practice or QOF achievement. Practice team and individual clinician behaviours, which appear poorly represented by routinely available indicators, are likely to account for substantial levels of variations in opioid prescribing. These may include established norms and procedures for reviewing prescribing, policies promoting continuity of care and adherence to best practice guidance on opioid prescribing.^54^ We further recognise the likely influence of wider system and social factors on prescribing, such as local prescribing formularies, legislation on controlled substances and media coverage.

Current prescribing trends signal a need for a concerted response to what is likely to be ineffective and harmful treatment for a large proportion of primary care patients. Once patients with coded cancer or substance dependence are accounted for, around 1 in 20 of the adult practice population is prescribed long-term opioids, a mean of 338 patients per practice. Nearly 1 in 100, or 59 adults per practice, are prescribed stronger opioids. Healthcare systems need to respond to two related problems: how to improve care for patients with chronic non-cancer pain; and how best to curb and reverse current trends in opioid prescribing, especially of stronger opioids.

One response is to recognise and manage chronic pain as a long-term condition which requires coordinated care including the formulation of care plans agreed between professionals and patients, effective non-pharmacological and self-management strategies, and ongoing active monitoring. We have demonstrated that it is feasible to identify patients within primary care based on prescribing records, potentially stratifying those at highest risk of stepping up to stronger opioid treatment as a priority group. These patient groups may extend beyond those with recognised and more severe chronic non-cancer pain. Patients with multimorbidity and polypharmacy represent the most obvious and pressing target for ‘deprescribing’.^55^ While we would not advocate non-negotiated reductions of regular modest prescriptions of weaker opioids, patients should be alerted to potential risks of continuing treatment at medication reviews.

Another response is to reduce initiation of opioid prescribing. Given the accumulating evidence about their harms, reversing the current trend in opioid prescribing is likely to benefit a substantial at-risk population. There are at least two evidence-based approaches to address this at practice level. First, audit and feedback is effective at changing professional practices.^56^ Its effects may be relatively modest but audit and feedback can be conducted efficiently using routinely recollected data and its effects may be enhanced by targeting higher baseline non-compliance with standards (eg, higher prescribing practices) and incorporating action plans for practices. Second, computerised prompts triggered by combinations of patient characteristics associated with stepping up to stronger opioids could alert clinicians to reconsider opioid prescribing at the point of care. Computerised clinical decision support is more likely to be effective if clinicians are required to supply a reason for over-riding advice.^57^

There is a growing consensus on best practice when considering or initiating opioids.^58–60^ This includes recognising and dealing with psychosocial aspects of pain, managing patient expectations about the degree of pain relief likely to be achievable, starting with a therapeutic trial and an agreement to stop or reduce opioids when they do not work, and recording care plans agreed with patients to guide all subsequent prescribers in maintaining the plan. GPs should avoid escalation above 120 mg morphine-equivalent daily equivalent and refer to pain management services if this seems likely.^60^

Strategies to bring about large-scale change in healthcare are unlikely to succeed if they fail to address multiple barriers and enablers across different levels of healthcare systems.^61^ Therefore, these types of above responses need to be coupled with educational campaigns to challenge or change professional and patient beliefs and expectations about opioid prescribing, drawing on lessons learned so far from strategies to reduce inappropriate prescribing of medicines such as antibiotics and minor hypnotics.^62^

Future research efforts should address strategies to reduce opioid prescribing which target patients and clinicians. There is little evidence on the effectiveness of interventions to reduce opioid prescribing in patients with chronic non-cancer pain.^63^ Non-pharmacological alternatives to pain management may be modestly beneficial but require further development and evaluation.^64^ There are parallel needs to further understand practice-level variation and evaluate interventions primarily aimed at clinicians to discourage initiation or continuation of opioid prescribing.
